# Supplementary material for: Keratin 8/18a.1 Expression Influences Embryonic Neural Crest Cell Dynamics and Contributes to Postnatal Corneal Regeneration in Zebrafish
Source: Cells. 2024 Sep 2;13(17):1473. doi: 10.3390/cells13171473 (PMC11394277; doi:10.3390/cells13171473)
Supplement: Supplementary file 1 [file cells-13-01473-s001.zip › cells-2819503-supplementary.pdf]

Supplemental figure S1

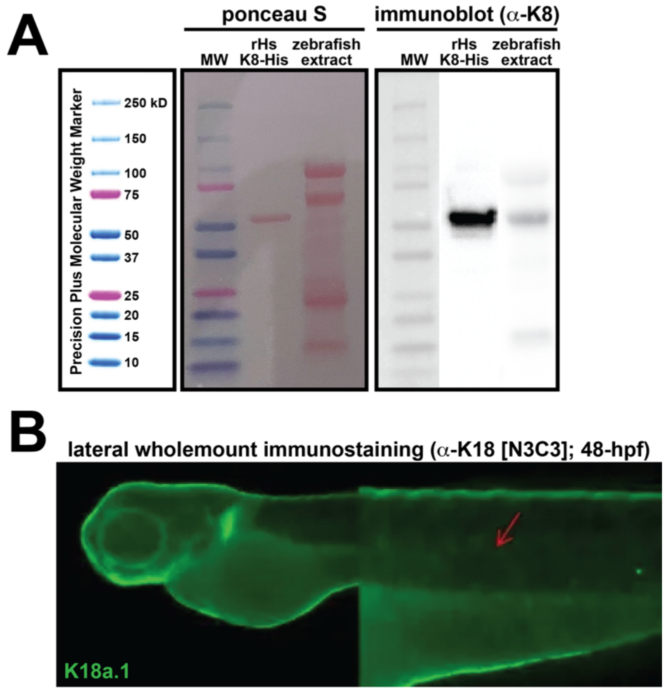

Supplemental Figure S1. Confirmation of antibody specificity. A) His-tagged recombinant human cytokeratin 8 protein (rHsK8-His; NBP2-23166; Novus Biologicals, Centennial, CO, United States) and a 48-hpf zebrafish whole embryo extract were denatured and separated by SDS PAGE, transferred to a polyvinylidene difluoride (PVDF) membrane (ThermoFisher Scientific, Waltham, MA, United States) and blotted with a mouse monoclonal antibody against cytokeratin 8 (1:500; GTX34663; GeneTex, Irvine, CA, United States). The primary antibody was detected using a goat anti-mouse HRP-linked secondary antibody (#7076S; Cell Signaling, Danvers, Massachusetts, United States), and the blot was subsequently developed using the Pierce Enhanced Chemiluminescence (ECL) Western Blotting Substrate kit (ThermoFisher). The results of staining with ponceau S are shown to document the pattern of the protein bands in each sample following transfer (*left panel*). A band of the expected molecular weight is observed in both samples (*right panel*). **B)** Immunohistochemical analysis (whole mount) of a 48-hpf zebrafish embryo, using a rabbit polyclonal antibody against cytokeratin 18 [(N3C3); GTX112978; GeneTex) at 1:200 dilution. This data image can be referenced on the GeneTex product page for the anti-K18 (N3C3) antibody and is provided as validation of the antibody specificity for zebrafish.
